# Supplementary material for: Structural and Electrical Investigation of Cobalt-Doped NiOx/Perovskite Interface for Efficient Inverted Solar Cells
Source: Nanomaterials (Basel). 2020 Apr 30;10(5):872. doi: 10.3390/nano10050872 (PMC7279223; doi:10.3390/nano10050872)
Supplement: Supplementary file 1 [file nanomaterials-10-00872-s001.pdf]

SUPPORTING INFORMATION

# Structural and electrical investigation of Cobalt doped $NiO_x$/perovskite interface for efficient inverted solar cells

**Zahra Rezay Marand,[a,b] Ahmad Kermanpur,[b] Fathallah Karimzadeh,[b] Eva M. Barea,[a] Ehsan Hassanabadi,[a,c] Elham Halvani Anaraki,[b] Beatriz Julian-Lopez,[a] Sofia Masi,[a,*] and Iván Mora-Seró[a,*]**

*Corresponding authors: masi@uji.es, sero@uji.es

[a] *Institute of Advanced Materials (INAM), Universitat Jaume I, Av. Sos Baynat, s/n, 12071 Castelló, Spain*

[b] *Department of Materials Engineering, Isfahan University of Technology, Isfahan 84156-83111, Iran*

[c] *Textile Engineering Department, Textile Excellence & Research Centers, Amirkabir University of Technology, Tehran, Iran*

**Table S1.** Grain sizes (nm) of the pure $NiO_x$ and different percentage Co doped nanoparticles, obtained from XRD measurement using the Debye-Scherrer formula $D_{XRD} = 0.89\ \lambda / \beta \cos\theta$, where $\lambda$ is the incident X-ray wavelength (1.54 A), $\theta$ is the Bragg diffraction angle, $\beta$ is the radian-based full width at half-maximum (FWHM) intensity of the peaks belonging to the XRD patterns in Figure 3a.

| | $NiO_x$ | 0.75mol % | 1mol % | 1.25mol % | 2.5mol % | 5mol % |
|---|---|---|---|---|---|---|
| Grain size | 17.1±0.4 | 12.4±0.3 | 11.9±0.2 | 12.1±0.2 | 12.3±0.3 | 11.5±0.2 |

a)

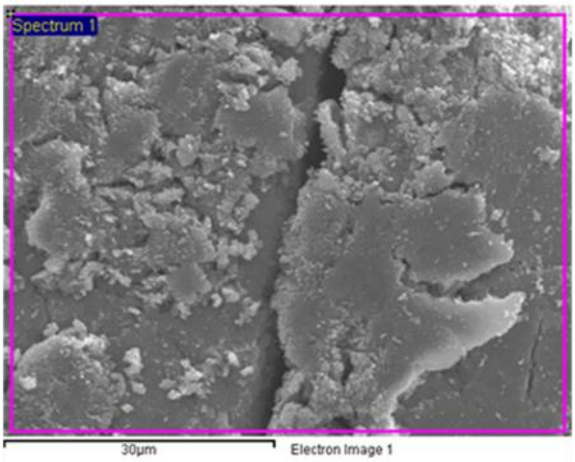


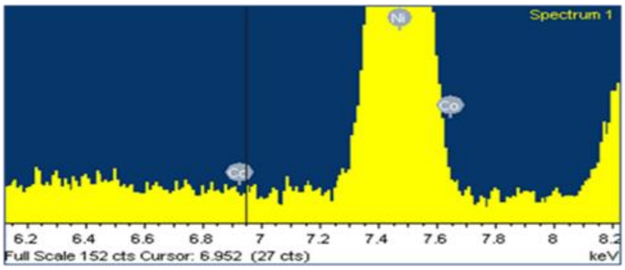


| Element | Weight% | Atomic% | Compd% | Formula |
|---|---|---|---|---|
| Co K | 0.00 | 0.00 | 0.00 | CoO |
| Ni K | 78.58 | 50.00 | 100.00 | NiO |
| O | 21.42 | 50.00 | | |
| Totals | 100.00 | | | |

b)

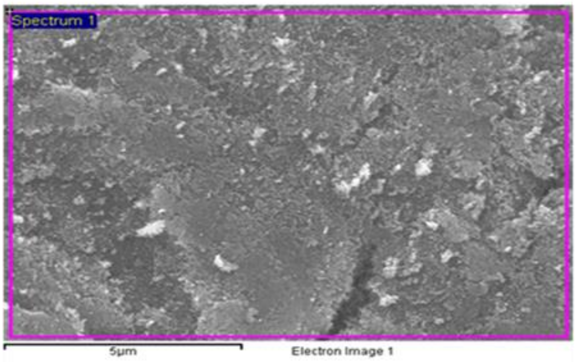


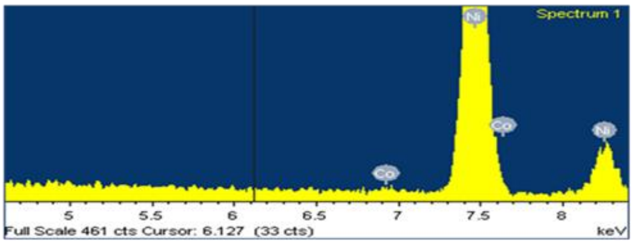


| Element | Weight% | Atomic% | Compd% | Formula |
|---|---|---|---|---|
| Co K | 0.55 | 0.35 | 0.70 | CoO |
| Ni K | 78.03 | 49.65 | 99.30 | NiO |
| O | 21.41 | 50.00 | | |
| Totals | 100.00 | | | |

c)

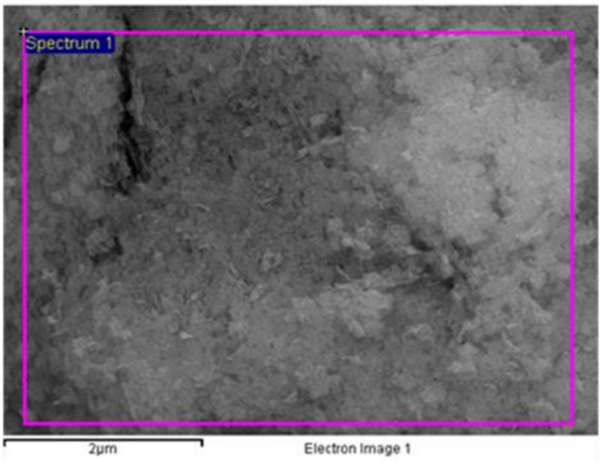


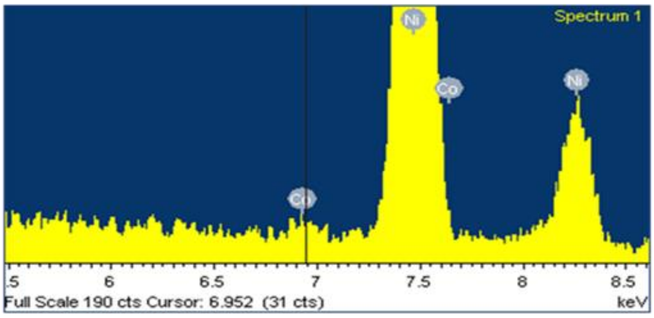


| Element | Weight% | Atomic% | Compd% | Formula |
|---|---|---|---|---|
| Co K | 0.92 | 0.58 | 1.17 | CoO |
| Ni K | 77.66 | 49.42 | 98.83 | NiO |
| O | 21.41 | 50.00 | | |
| Totals | 100.00 | | | |

d)

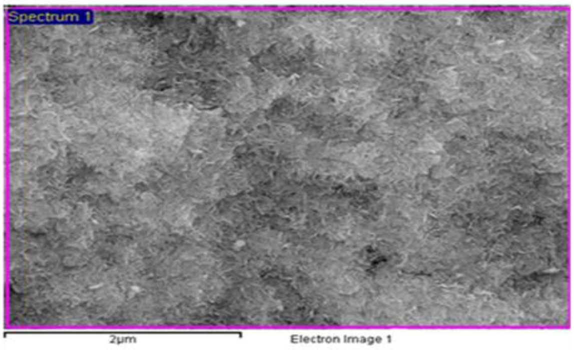


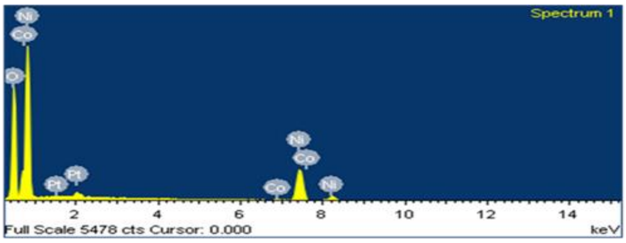


| Element | Weight% | Atomic% | Compd% | Formula |
|---|---|---|---|---|
| Co K | 1.09 | 0.69 | 1.38 | CoO |
| Ni K | 77.50 | 49.31 | 98.62 | NiO |
| O | 21.41 | 50.00 | | |
| Totals | 100.00 | | | |

e)

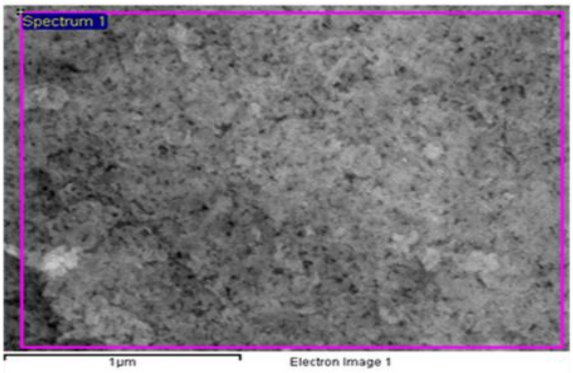


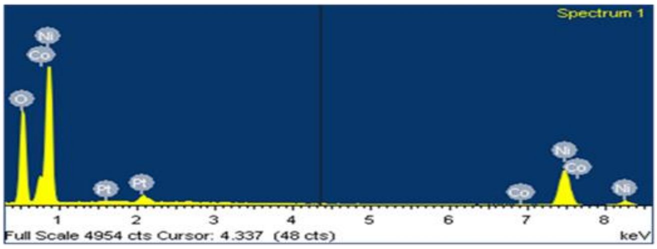


| Element | Weight% | Atomic% | Compd% | Formula |
|---|---|---|---|---|
| Co K | 2.09 | 1.33 | 2.66 | CoO |
| Ni K | 76.49 | 48.67 | 97.34 | NiO |
| O | 21.41 | 50.00 | | |
| Totals | 100.00 | | | |

f)

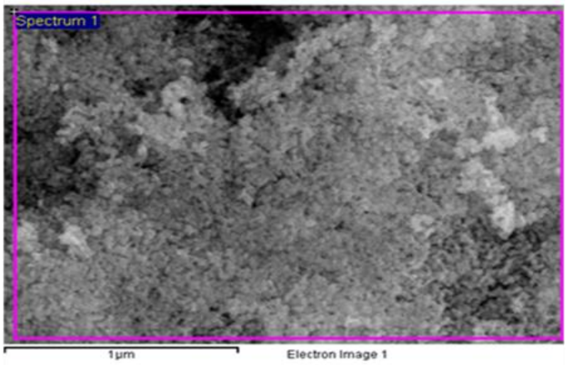


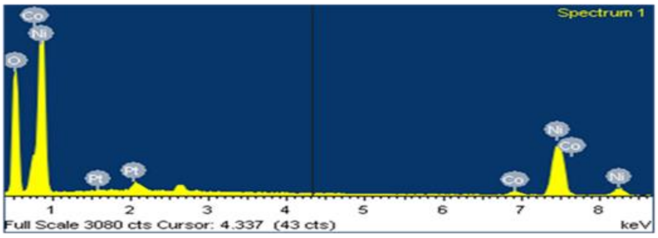


| Element | Weight% | Atomic% | Compd% | Formula |
|---|---|---|---|---|
| Co K | 3.91 | 2.48 | 4.97 | CoO |
| Ni K | 74.68 | 47.52 | 95.03 | NiO |
| O | 21.41 | 50.00 | | |
| Totals | 100.00 | | | |

**Figure S1.** EDS spectra for $NiO_x$ and $Co\text{-}NiO_x$ nanoparticles and quantitative analysis for each element (Ni, Co, O). a) $NiO_x$. b) 0.75 mol% $Co\text{-}NiO_x$. c) 1 mol% $Co\text{-}NiO_x$. d) 1.25 mol% $Co\text{-}NiO_x$. e) 2.5 mol% $Co\text{-}NiO_x$. f) 5 mol% $Co\text{-}NiO_x$.

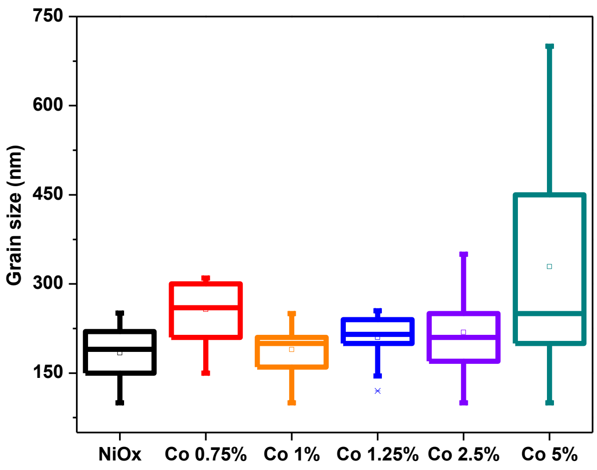


**Figure S2.** Perovskite grain size statistical distributions of the film deposited on the $NiO_x$ with and without Co doping at different percentage (from 0.75 mol% to 5 mol%). The grain size is calculated with the SEM ruler for 40 grains of each kind of sample.

**Table S2.** Intensity of the XRD perovskite peaks (counts) and ratio between the intensity of the peaks 110 (14.2 °) and 220 (28.5 °).

| substrate | (110) | (220) | (110)/(220) |
|---|---|---|---|
| $NiO_x$ | 563 | 288 | 1.95 |
| Co 0.75% | 699 | 285 | 2.45 |
| Co 1% | 584 | 276 | 1.11 |
| Co 1.25% | 630 | 287 | 2.19 |
| Co 2.5% | 549 | 282 | 1.95 |
| Co 5% | 396 | 226 | 1.75 |

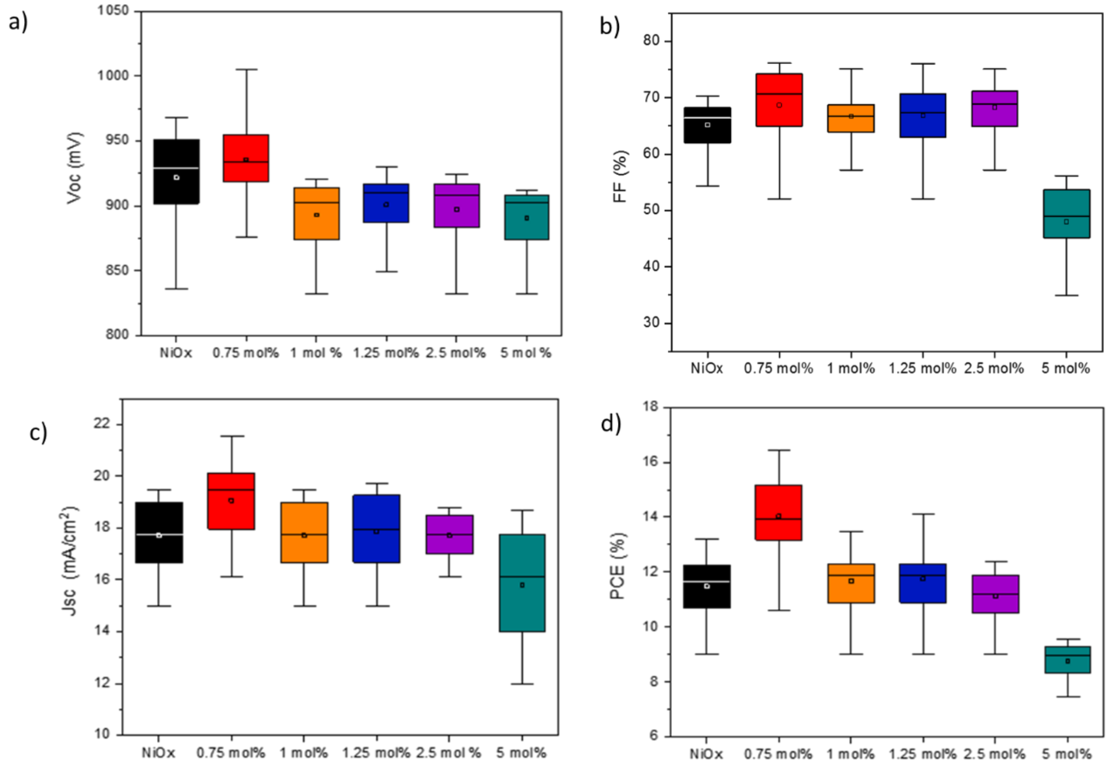


**Figure S3.** Statistical analysis of a) $V_{oc}$ b) FF, c) $J_{sc}$, and d) PCE of 80 perovskite solar cells fabricated with 0-75-5% mol% Co-$NiO_x$ and $NiO_x$.

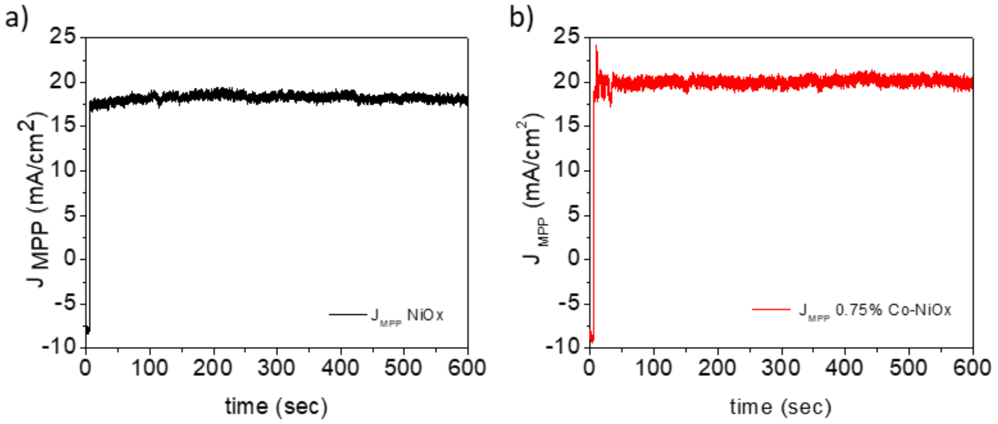


**Figure S4.** Recorded photocurrent at maximum power point $J_{MPP}$ of the champion devices in the case of control NiOx and 0.75 mol% Co-NiOx based cells.

| Table S3. Photoluminescence time decay parameters τ1 and τ2 and their average. | | | |
|---|---|---|---|
| | $\tau_1$ (ns) | $\tau_2$ (ns) | $\tau_{average}$ (ns) |
| $MAPbI_3$ on ITO | 2.3 | 33 | 32 |
| $MAPbI_3$ on $NiO_x$ | 1.6 | 20 | 20 |
| $MAPbI_3$ on 0.75 mol% Co-$NiO_x$ | 0.8 | 10 | 9.8 |
| $MAPbI_3$ on 1 mol% Co-$NiO_x$ | 0.9 | 9 | 8.5 |
| $MAPbI_3$ on 1.25 mol% Co-$NiO_x$ | 1.8 | 11 | 10.3 |
| $MAPbI_3$ on 2.5 mol% Co-$NiO_x$ | 2.1 | 14 | 12.5 |
| $MAPbI_3$ on 5 mol% Co-$NiO_x$ | 1.5 | 13 | 11.2 |

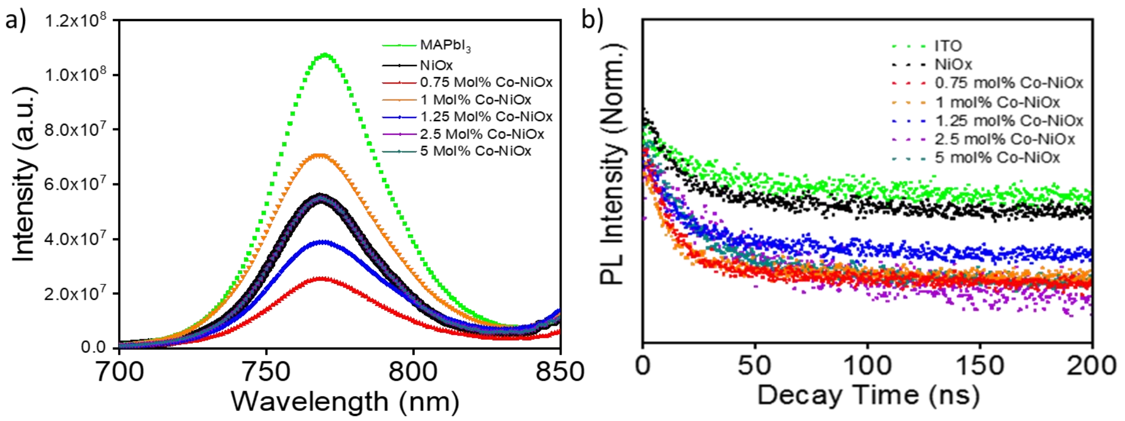


**Figure S5.** a) PL of $MAPbI_3$ films on top of $NiO_x$ and 0.75-5 mol % Co-$NiO_x$. The $MAPbI_3$ on top of glass was used as a reference measurement; b) Time-resolves PL of the corresponding samples.

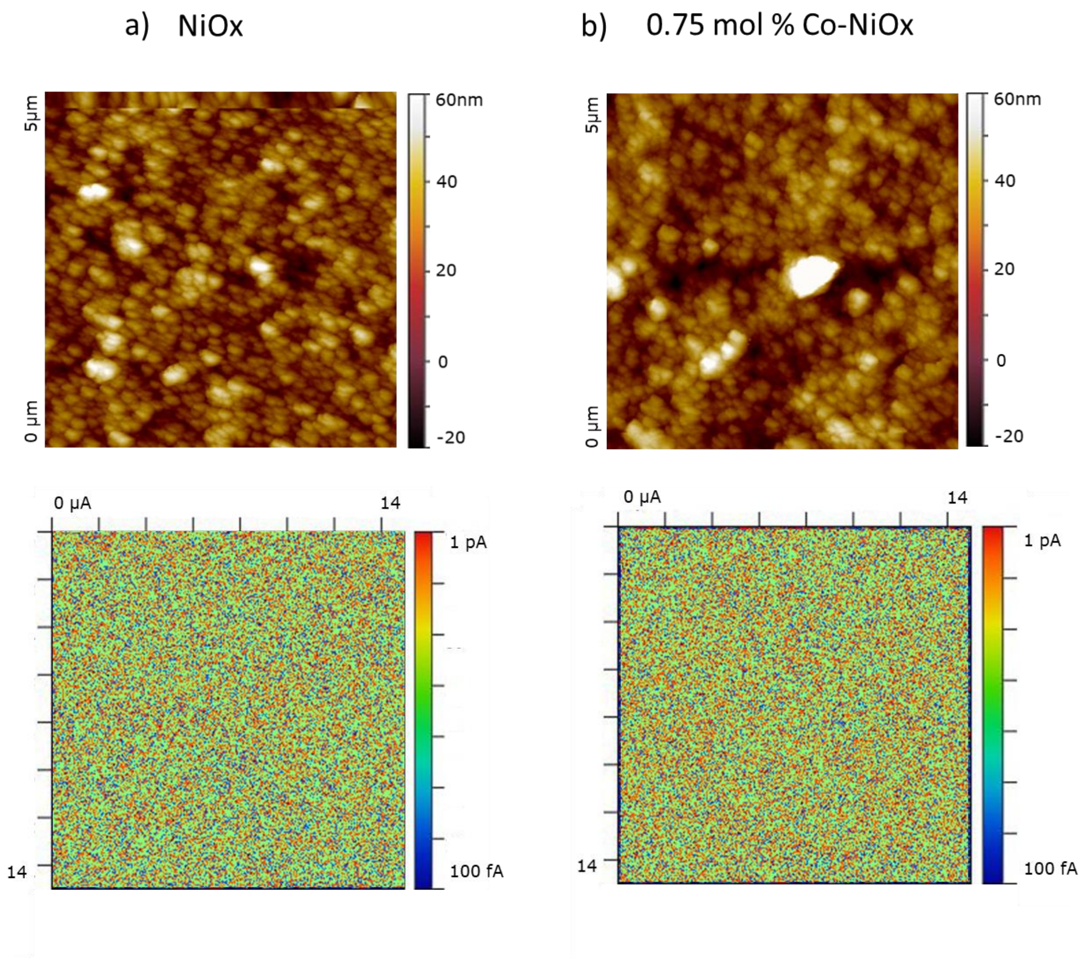


**Figure S6.** Conductive-AFM topographies on ITO substrates of a) pristine $NiO_x$ and b) 0.75 mol% Co-$NiO_x$.
